# RACIAL AND ETHNIC VARIATIONS IN THE EFFECTS OF KNOWING ABOUT ONE’S DIAGNOSTIC LABEL OF DEMENTIA ON SOCIAL SUPPORT

**DOI:** 10.1093/geroni/igad104.1564

**Published:** 2023-12-21

**Authors:** Takashi Amano, Cal Halvorsen, Seoyoun Kim, Addam Reynolds, Clara Scher, Yuane Jia

**Affiliations:** Rutgers University, Newark, New Jersey, United States; Boston College, Boston, Massachusetts, United States; Texas State University, San Marcos, Texas, United States; University of Southern California, Los Angeles, California, United States; Rutgers, The State University of New Jersey, New Brunswick, New Jersey, United States; Rutgers University, Newark, New Jersey, United States

## Abstract

Theoretically, knowing about one’s diagnostic label of dementia may increase a chance to receive necessary social support. However, empirical evidence especially regarding the racial/ethnic variations in the effects of diagnostic labeling on provision of social support is lacking. Data from the Health and Retirement Study (HRS, 2000-2018) were utilized to examine variations in the effects of knowing about a diagnostic labeling. A total of 7,192 person-year observations who had dementia were included in the analysis. Knowledge about a diagnostic label of dementia was measured by asking whether a doctor told the person that he/she had dementia. Formal and informal types of received social support were measured. Regression analyses with inverse probability weighting were performed. A moderating role of race/ethnicity was examined by conducting subgroup analyses. Knowing about a diagnostic label significantly increased likelihood of having more informal (OR = 1.58, p < 0.001, 95% CI = [1.34, 1.86]) and formal support (b = 0.09, p < 0.001, 95% CI = [0.05, 0.13]). The significant effect of a diagnostic label on formal support was not observed among non-Hispanic Black (b = 0.07, p = 0.086, 95% CI = [-0.01, 0.15]) or Hispanic (b = 0.07, p = 0.152, 95% CI = [-0.03, 0.16]) individuals. Results suggest that knowing about a diagnostic label of ADRD may increase informal and formal social support. However, the beneficial impacts may not be salient for non-Hispanic Black and Hispanic individuals. Strategies to maximize the beneficial effects of diagnostic labeling should be identified to address racial/ethnic disparities.

